# *Plasmodium falciparum* Histidine-Rich Protein 2 and 3 Gene Deletions in Strains from Nigeria, Sudan, and South Sudan

**DOI:** 10.3201/eid2702.191410

**Published:** 2021-02

**Authors:** Christiane Prosser, Karryn Gresty, John Ellis, Wieland Meyer, Karen Anderson, Rogan Lee, Qin Cheng

**Affiliations:** Australian Defence Force Malaria and Infectious Disease Institute, Brisbane, Queensland, Australia (C. Prosser, K. Gresty, K. Anderson, Q. Cheng);; Westmead Institute for Medical Research, Westmead, New South Wales, Australia (C. Prosser, W. Meyer);; University of Sydney, Sydney, New South Wales, Australia (C. Prosser, W. Meyer, R. Lee);; QIMR–Berghofer Medical Research Institute, Brisbane (K. Gresty, K. Anderson, Q. Cheng);; University of Technology Sydney, Sydney (J. Ellis);; Westmead Hospital, Westmead (W. Meyer);; Marie Bashir Institute for Infectious Diseases and Biosecurity, University of Sydney, Sydney (W. Meyer);; Centre for Infectious Diseases and Microbiology Laboratory Services, ICPMR, Westmead Hospital, Westmead (R. Lee)

**Keywords:** *Plasmodium falciparum*, imported malaria, *pfhrp2*, *pfhrp3*, rapid diagnostic tests, travelers’ health, molecular surveillance, parasites, Nigeria, Sudan, South Sudan

## Abstract

Deletion of histidine-rich protein genes *pfhrp2/3* in *Plasmodium falciparum* causes infections to go undetected by HRP2-based malaria rapid diagnostic tests. We analyzed *P. falciparum* malaria cases imported to Australia (n = 210, collected 2010–2018) for their *pfhrp2/3* status. We detected gene deletions in patients from 12 of 25 countries. We found >10% *pfhrp2-*deletion levels in those from Nigeria (13.3%, n = 30), Sudan (11.2%, n = 39), and South Sudan (17.7%, n = 17) and low levels of *pfhrp3* deletion from Sudan (3.6%) and South Sudan (5.9%). No parasites with *pfhrp2/3* double deletions were detected. Microsatellite typing of parasites from Nigeria, Sudan, and South Sudan revealed low relatedness among gene-deleted parasites, indicating independent emergences. The gene deletion proportions signify a risk of false-negative HRP2-RDT results. This study’s findings warrant surveillance to determine whether the prevalence of gene-deleted parasites justifies switching malaria rapid diagnostic tests in Nigeria, Sudan, and South Sudan.

During 2000–2015, global malaria incidence and death rates were reduced by more than half (*1*). Malaria control efforts are credited with increasing life expectancy by 5% globally and by 12.3% in sub–Saharan Africa, where ≈90% of the disease burden is located (*2*). Gains in malaria control have been attributed primarily to the implementation of key intervention measures including insecticide-treated netting, indoor residual spraying, combination medicines, and diagnostic tests. Malaria decline has been more gradual, or has stalled, in endemic regions with limited access to these interventions (*3*).

Rapid diagnostic tests (RDTs) are recommended, and have become essential, for malaria case management in many regions because they meet the challenges for remote and low-resource settings. These tests are affordable, easy to transport and store, and less skill- and resource-demanding than microscopy, but they offer comparable sensitivity to quality microscopy (*4*). These RDTs are used, along with Giemsa-stained blood films, for diagnosis of imported malaria in pathology laboratories in Australia (including those of the Australian Defence Force) (*5*). A preliminary diagnosis using RDTs is made and the diagnosis confirmed by stained thick and thin films. False-negative tests from RDTs will result in delayed treatment, which may affect the patient’s clinical outcomes.

Histidine-rich protein 2 (HRP2)–based RDTs are largely preferred for detecting *P. falciparum* malaria because of their superior sensitivity and heat-stability profile over *Plasmodium* lactate dehydrogenase (pLDH) or aldolase (*6*). HRP2-based tests detect the HRP2 antigen (and, to a lesser extent, HRP3, because of cross-reactivity) at levels as low as ≈1 ng/mL blood; however, in practice, the detection limit of HRP2-based tests is reportedly comparable with that of quality microscopy (≈200 parasites/μL) (*7*). This level is adequate for case management but much less sensitive than molecular methods. RDTs have been reported to have failed to detect a substantial proportion of asymptomatic infections (*8*).

Parasite deletion of the genes *pfhrp2* and *pfhrp3* has been implicated in false-negative results using HRP2-based RDTs. There are recent reports of *pfhrp2*-deleted parasites in several countries in Africa (*9*–*15*), as well as India (*16*), China and Myanmar (*17*), and countries in South America, including Peru (*18*). Single *pfhrp2* gene deletions represent an increased risk for RDT failure, especially in cases of low parasitemia or inferior RDTs (*19*). In the instance of a double deletion of *pfhrp2* and *pfhrp3,* the parasite is undetectable with HRP2-based RDTs (*20*). Because RDTs are the mainstay diagnostic tool for many endemic countries, loss of effectiveness constitutes a public health emergency and poses a major challenge to *P. falciparum* control and elimination efforts. For countries reliant on RDTs, gene-deletion prevalence data are needed to inform case management policy.

The World Health Organization has estimated a threshold of 5% of parasites lacking HRP2 as the point at which false negatives from lack of antigen expression would likely exceed the rate of false negatives observed using alternative RDTs and, as such, the point at which HRP2-based tests are no longer recommended for that location (*21*). Therefore, surveillance is critical to estimate whether the prevalence of parasites with gene deletions has reached the threshold for switching RDTs and is recommended to focus primarily on locations or nearby locations where gene deletions have been detected. Imported cases of malaria are a resource to detect gene deletions in countries of origin and the outcomes can prompt large-scale surveillance.

When case management policies for imported malaria are developed, regional *pfhrp2*/*pfhrp3* deletion levels should also be considered. The lack of clarity regarding the status of many endemic regions has fueled concern on the part of physicians. In settings where only RDTs are used for diagnosis, laboratories need to be aware of the possibility of false negatives when testing samples with >5% rate of HRP2 deletion. Consequently, we investigated the *pfhrp2*/*pfhrp3* status of malaria cases imported from travelers, immigrants, and refugees entering Australia to identify evidence for *pfhrp2* and *pfhrp3* deletions in *P. falciparum* from malaria-endemic countries.

## Methods

### Sample Collection and DNA Extraction

Malaria cases in Australia require that a blood sample be sent to a regional reference laboratory for confirmation and storage. We determined *Plasmodium* spp*.* infection and species by microscopy (Giemsa-stained thick and thin smears) and confirmed them by PCR (*22*) at the New South Wales Health Pathology Parasitology Laboratory at Westmead Hospital (Westmead, New South Wales, Australia). We aliquoted whole blood from archived *P. falciparum*–positive samples from imported malaria cases (n = 210) and recorded deidentified patient information. We extracted genomic DNA from whole blood using QIAamp mini DNA kits (QIAGEN, https://www.qiagen.com) according to the manufacturer’s directions. We assessed DNA quality by subjecting DNA to agarose gel electrophoresis. We measured DNA concentrations by spectrophotometric analysis using a Nanodrop Spectrophotometer ND-1000 (Thermo Fisher, https://www.thermofisher.com) at 260 nm and 280 nm. We included genomic DNA from *P. falciparum* laboratory reference strains in each PCR assay as experimental controls for various *pfhrp2/pfhrp3* deletion statuses: 3D7 (*pfhrp2* +/*pfhrp3* +), HB3 (*pfhrp2* +/*pfhrp3* −), 3BD5 (*pfhrp2* −/*pfhrp3* −), Dd2 (*pfhrp2* −/*pfhrp3* +), and D10 (*pfhrp2* −/*pfhrp3* +). We stored samples at −20°C before use.

### Characterization of *pfhrp2* and *pfhrp3*

We investigated the status (presence/absence) of *pfhrp2* (PlasmoDB gene ID Pf3D7_0831800) and *pfhrp3* (PlasmoDB gene ID Pf3D7_1372200) genes by amplifying across exon 1–exon 2 and exon 2, as previously described (*10*; Appendix Table). Samples were considered to contain the *pfhrp2*- or *pfhrp3*-deleted parasites when there was a negative PCR result for exon 1 or exon 2 of the gene, or both, along with a positive PCR amplifying all 3 single-copy reference genes: merozoite surface protein 1 (*pfmsp1*), merozoite surface protein 2 (*pfmsp2*), and glutamate-rich protein (*pfglurp*). The use of the comparable single copy reference gene assays as a DNA quality control has been observed in several studies reporting *P. falciparum* with and without deletions to show a concordant limit of detection when the genes are present (*23*).

### Rapid Diagnostic Testing

We used SD Bioline (Standard Diagnostics, https://www.globalpointofcare.abbott) HRP2-based malaria RDTs according to the manufacturer’s instructions to test thawed whole blood samples that had been determined to contain *P. falciparum* with gene deletions (when whole blood was available). We performed additional tests on *pfhrp2/pfhrp3* positive and negative samples at various parasite densities, and we conducted comparative tests using BinaxNOW (Inverness Medical Binax, https://www.globalpointofcare.abbott) and Carestart (AccessBio, https://accessbiodiagnostics.net) HRP2-based malaria RDTs.

### Microsatellite Analysis

We conducted microsatellite analysis as described elsewhere (*10*). In brief, for each sample originating from Sudan, South Sudan, or Nigeria, we analyzed 7 neutral microsatellite markers (TA1, PolyA, PfPK2, TA109, 2490, 313, and 383). We amplified markers per PCR conditions and primers listed (Appendix Table). We sized amplicons using an ABI 3100 Genetic Analyzer (Applied Biosystems, https://www.thermofisher.com). We scored alleles manually using Peak Scanner Software version 1.0 (Applied Biosystems), including a minimum peak height of 300 relative fluorescence units (Appendix Figure 1). To exclude artifactual stutter peaks (likely polymerase slippage on extended tandem repeats, which are frequent in *Plasmodium* genomes), we disregarded peaks less than one third of the predominant peak (*24*).

### Genetic Diversity Phylogenetic Analysis

We produced a predominant haplotype for each sample based on the sizes of the 7 microsatellite markers. We used PHYLOViZ software (*25*) using a minimum spanning tree approach to compare the genetic diversity and genetic relatedness of the Sudan, South Sudan, and Nigeria cohorts within this study and to compare with parasites from Eritrea and Peru (haplotypes characterized in a previous study [*10*]). We standardized values for the microsatellite marker sizes against the *P. falciparum* 3D7 reference strain. We used FSTAT to calculate microsatellite allele frequencies at each locus, average number of alleles, and expected and observed heterozygosity (*26*).

## Results

### Patient Data Analysis

This study included parasite samples from persons from 25 countries, with most (194/210) originating from countries in Africa. A large proportion of the patient cohort traveled to Australia from Nigeria (n = 30) or Sudan (n = 39); for all other countries of origin, n<20. The clinical state, when known, was predominantly symptomatic travelers who came to the hospital; however, the cohort included >15 potentially asymptomatic samples collected during refugee screening (n = 8 within the cohort from South Sudan). The study population was composed of 149 male patients, 53 female patients, and 8 patients with unknown gender; age range was 6 months to 79 years at the time of infection (median age 42 years). Of the samples collected, 75.2% had a parasitemia ranging from 0.01% (≈500 parasites/μL) to 30.1% (1,505,000 parasites/μL), with a mean of 1.34 ± 3.00%, 67,000 parasites/μL; 24.8% had a parasitemia <0.01%. Only 48% of patients (when reported) had used chemoprophylaxis (doxycycline, artemether/lumefantrine, or mefloquine), and instances of concurrent conditions were low (reported in <5% of cases, most commonly dengue fever; Appendix Table 2). 

### Presence/Absence of *pfhrp2* and *pfhrp3*

**Figure 1:**
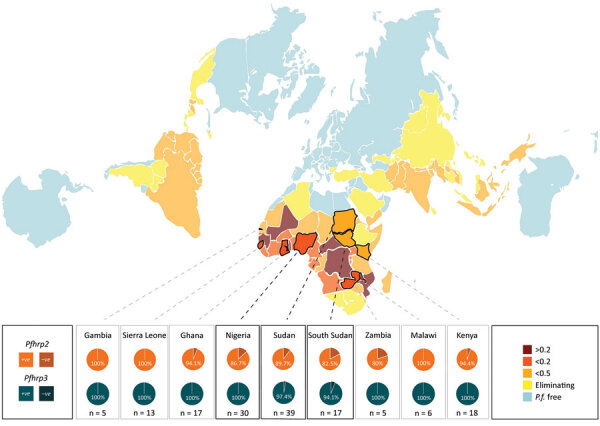
Summary of *pfhrp2* and *pfhrp3* deletion key results showing *pfhrp2* and *pfhrp3* deletion results for *Plasmodium* spp. isolates, by parasite country of origin (where n>4), Australia. National *P. falciparum* endemicity depicted is measured as population-weighted mean *P. falciparum* infection rate of children 2–10 years of age, using data available from the Malaria Atlas Project (http://www.map.ox.ac.uk). Data were mapped using the AuthaGraph world map projection to more truthfully visualize the potential paths of dissemination and adjacency of various endemic zones, as this is considered the most accurate representation of land proportions and relative orientations (https://hrcak.srce.hr/185867). *P.f.*, *P. falciparum*.

We observed *pfhrp2* or *pfhrp3* deletion (together with positive *pfmsp1*, *pfmsp2*, and *pfglurp* results) in 24 of 210 parasite samples from 12 of 25 countries of origin (Table 1). Results from assays amplifying exon 2 of *pfhrp2* and *pfhrp3* matched the findings from assays amplifying across exon 1–2, suggesting whole rather than partial gene deletion. We observed *pfhrp2*-deleted parasites in 3 samples from Nigeria (3/30, 10%), 4 samples from Sudan (4/39, 10.26%), and 4 samples from South Sudan (4/17, 17.65%) (Figure 1). We observed a single sample with *pfhrp2*-deletion in specimens originating from Ghana (1/17, 5.88%), Kenya (1/18, 5.55%), Mali (1/3, 33.33%), Togo (1/1, 100%), and Zambia (1/5, 20%). We found 3 samples (3/27) of unknown African origins to be *pfhrp2-*deleted.

**Table 1:** Summary of *pfhrp2/pfhrp3* gene deletion screening results showing *pfhrp2/pfhrp3* status for *Plasmodium* spp. isolates, by parasite country of origin, Australia*

Source	Country/strain name	No. cases	pfhrp2/pfhrp3 status, no. (%)			% Symptomatic	% Refugee
			+/−	−/+	−/−		
Africa	Cameroon	3	0	0	0	66.6	33.3
	Gambia	5	0	0	0	100	0
	Ghana	17	0	1 (0.06)	0	100	0
	Ivory Coast	2	0	0	0	100	0
	Kenya	18	0	1 (0.06)	0	100	0
	Madagascar	1	0	0	0	100	0
	Malawi	6	0	0	0	33.3	66.6
	Mali	3	0	1 (33.3)	0	100	0
	Nigeria	30	0	4 (13.3)	0	100	0
	Sierra Leone	13	0	0	0	92.3	7.7
	South Africa	2	0	0	0	100	0
	South Sudan	17	1 (5.9)	3 (17.6)	0	52.9	47.1
	Sudan	39	1 (2.6)	4 (10.3)	0	100	0
	Sumatra	2	1 (50)	0	0	100	0
	Togo	1	0	1 (100)	0	100	0
	Tanzania	4	1 (25)	0	0	100	0
	Uganda	2	0	0	0	100	0
	Zambia	5	0	1 (20)	0	100	0
	Zimbabwe	1	0	0	0	100	0
	Unknown†	27	0	3 (11.1)	0	100	0
Asia	Cambodia	1	0	0	0	100	0
	India	3	0	0	0	100	0
	Indonesia	1	0	0	0	100	0
	Papua New Guinea	5	0	0	0	100	0
	Thailand	1	0	0	0	100	0
South America	Peru	1	1 (100)	0	0	100	0
Laboratory strains	3D7	1	0	0	0	NA	NA
	3BD5	1	0	0	1 (100)	NA	NA
	D10	1	0	1 (100)	0	NA	NA
	Dd2	1	0	1 (100)	0	NA	NA
	HB3	1	0	1 (100)	0	NA	NA

*NA, not applicable. †Cases in which clinical notes state African origins but do not specify a country.

We observed a single sample with *pfhrp3* deletion per origin in parasites from Sudan (1/39, 2.56%), South Sudan (1/17, 5.88%), Tanzania (1/4, 25%), Sumatra (1/2, 50%), and Peru (1/1, 100%). No parasites were observed to have both the *pfhrp2* and *pfhrp3* gene deletion.

### Rapid Diagnostic Test Results

We tested 20 gene deletion blood samples with HRP2 RDTs (18 *pfhrp2* deleted, 2 *pfhrp3* deleted). Of these, 16 samples produced a positive Pf band using HRP2-based SD BioLine malaria RDTs (14 *pfhrp2* deleted, 2 *pfhrp3* deleted). Of the 16 gene deletion parasites detected by HRP2 RDT, 10 samples had a parasitemia >1000/μL. Four of 18 *pfhrp2-*deleted parasites failed to be detected by HRP2 RDTs; 3 of these 4 cases had a parasitemia level <500/μL (Table 2). Only 9 of 20 samples gave a positive pan band; 8 of the 9 had a parasitemia level >2,000/μL.

**Table 2:** Assessment of HRP2-based SD BioLine RDT for *pfhrp2/pfhrp3* deletion genotypes for *Plasmodium* spp. isolates, by parasite country of origin, Australia*

Country	Sample ID	Collection year	Parasitemia, % erythrocytes	Parasites/μL blood	Genotype, *pfhrp2/pfhrp3*	BioLine RDT†	
						Pan	*Pf*
Sudan	BDA1	2016	0.16	8,000	+/+	1	2
Sudan	BDA2	2016	0.79	39,500	–/+	1	3
Sudan	BDA3	2016	0.3	15,000	–/+	0	1
Sudan	BDA4	2016	0.02	1,000	–/+	0	1
Sudan	BDA37	2014	<0.01	NA	–/+	0	0
South Sudan	BDD4	2018	1.24	62,000	+/–	1†	3
South Sudan	BDC98	2018	0.5	25,000	–/+	1	1
South Sudan	BDB94	2017	0.5	25,000	–/+	1	1
South Sudan	BDB99	2017	<0.01	NA	–/+	0	1†
Nigeria	BDA24	2015	1.1	55,000	–/+	0	1
Nigeria	BDA92	2012	4	200,000	–/+	1	1
Nigeria	BDA91	2012	2.5	125,000	–/+	1	1
Nigeria	BDB31	2011	0.08	4,000	–/+	0	0
Kenya	BDA42	2014	0.12	6,000	–/+	2	1
Kenya	BDB19	2011	<0.01	NA	+/+	0	3
Ghana	BDA5	2016	<0.01	NA	–/+	1‡	1‡
Tanzania	BDA28	2015	<0.01	NA	–/+	0	1
Zambia	BDA31	2015	0.2	10,000	–/+	0	1
Togo	BDA50	2014	0.27	13,500	–/+	0	1
Peru	BDA52	2014	0.4	20,000	+/–	1	3
Papua New Guinea	BDB6	2012	2.81	140,500	+/+	1‡	2
Papua New Guinea	BDB5	2012	<0.01	NA	+/+	1‡	1
Africa	BDA99	2012	0.46	23,000	+/+	0	3
Africa	BDA90	2012	1.2	60,000	+/+	0	3
Africa	BDA100	2012	<0.01	NA	+/+	0	1
Africa	BDB11	2011	<0.01	NA	–/+	0	0
Africa	BDB46	2010	0.01	500	–/+	0	0

*Gray shading signifies a negative result with both the pan-pLDH and pfHRP2 antigen tests on the RDT. Africa indicates unknown country of origin. ID, identification; NA, not applicable; pan, pan–*Plasmodium* spp.; *Pf*, *P. falciparum*; pfHRP2, *Plasmodium falciparum* histidine-rich protein 2; pLDH, *Plasmodium* lactate dehydrogenase; RDT, rapid diagnostic test. †0–4 result scored by World Health Organization guidelines. ‡Sample results matched outcomes using CareStart Malaria RDT, whereas parasites were not detected by BinaxNOW RDT (Appendix Figures 2, 3).

### Microsatellite Analysis

**Figure 2:**
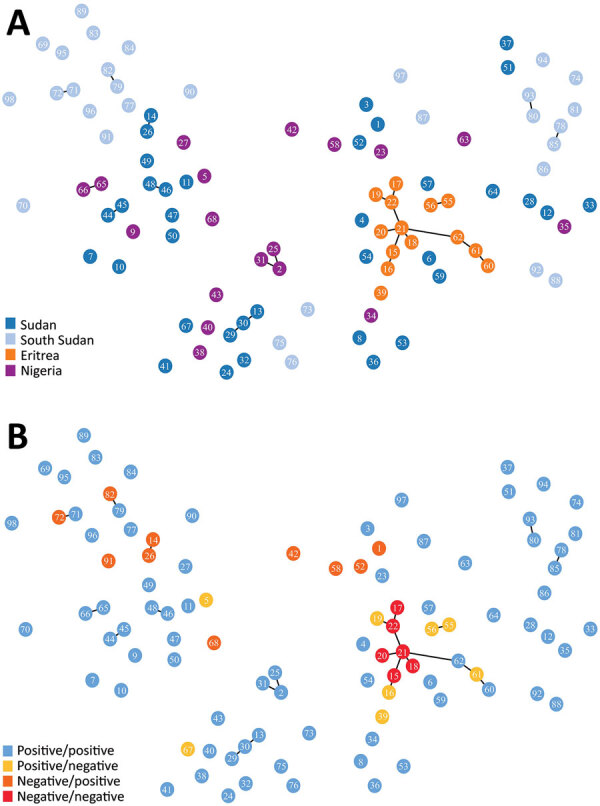
Minimum spanning tree of microsatellite allelic data showing genetic relatedness of *Plasmodium falciparum* populations from Sudan, South Sudan, Nigeria, and Eritrea (A), and *pfhrp2* and *pfhrp3* deletion status of haplotypes (B) (positive: gene present; negative: gene absent). Numbered circles represent specific haplotypes. Plots were generated using PHYLOViZ software (*25*) with a cutoff value of 2 (minimum differences for 2 microsatellite loci) depicted as lines connecting haplotypes and a cutoff value of 3 depicted as haplotype circle arrangements/proximities.

We amplified and scored 7 microsatellite loci for each sample from Sudan, South Sudan, and Nigeria (n = 86), finding 88 unique haplotypes. Two samples shared a haplotype, and we observed 2 instances of multiple haplotype infection. All 7 microsatellite markers were found to be polymorphic. We found a mean of 11 alleles per locus, a range from 6 (microsatellite markers TA109 and 2490) to 16 (microsatellite marker 383) distinct alleles. We found the genetic relatedness of *P. falciparum* populations to correspond weakly with country of origin (represented by small clusters of 2–3 haplotypes), as compared with the population structure of parasites from Eritrea (Figure 2, panel A). Unlike large clustering of *pfhrp2/3*-deleted parasites in Eritrea, *pfhrp2-* or *pfhrp3-*deleted parasites within the cohorts from Sudan, South Sudan, and Nigeria were not found to be more closely related to each other than to *pfhrp2/pfhrp3*-positive parasites within their cohort (Figure 2, panel B). The expected heterozygosity of populations (by country and by deletion status) did not exceed the observed heterozygosity for any cohort.

## Discussion

Increasing availability and use of HRP2-based malaria RDTs in Africa has been pivotal to improving case management over the past 20 years (*27*). Evaluation of compliance to RDT outcomes in sub-Saharan Africa found that protocols often varied among healthcare workers, particularly in the case of negative RDT results (*28*). Increased rates of RDT false-negative results may undermine confidence in adherence to World Health Organization guidelines (*29*) and would threaten the recent gains in malaria control.

Several countries relying on HRP2-based malaria RDTs lack molecular data on parasite *pfhrp2* and *pfhrp3* deletion. Sudan and South Sudan had no previously reported data regarding *pfhrp2* and *pfhrp3* status. We observed the presence of both *pfhrp2* and *pfhrp3*-deleted parasites in this study, although no double deletions were detected. The presence of these parasites is not altogether unexpected given the low level of *pfhrp2*- and *pfhrp3*-deleted parasites previously found in natural *P. falciparum* populations (*30*) and the presence of these parasites in neighboring endemic regions (*10*). The levels of *pfhrp2* deletion raise concerns: 10.3% observed from Sudan (mean collection date 2016), and 17.5% from South Sudan (mean collection date 2017–2018) (Figure 1). Mathematical modeling predicts rapid (≈3 years) selection for widespread *pfhrp2-*deletion within a population subjected to HRP2-based RDT use, with a baseline *pfhrp2-*deletion level lower than what we observed in parasites originating from Sudan and South Sudan (*31*). In addition, this region of Africa experiences a great deal of human migration (*32*,*33*), notably in Sudan’s neighbor Eritrea (where *pfhrp2*/*pfhrp3* double deletion parasites are prevalent [*10*]), increasing the risk for deletion-parasite dissemination.

Samples originating from Nigeria (n = 30) were collected during 2011–2015 (1 sample from 2011 was, to our knowledge, the earliest reported *pfhrp2-*deleted parasite from Nigeria); however, the proportion of *pfhrp2*-deleted parasites observed (13.3%) is similar to the 17% observed in a 2019 study of contemporary parasites from Nigeria (likewise finding no double deletion) (*14*). Countries in western Africa, such as Nigeria, often make use of exclusively HRP2-based RDTs (no pan–*Plasmodium* spp. antigen target); because reliance on *P. falciparum*–only RDTs would further exacerbate the public health consequences of *pfhrp2*/*pfhrp3-*deleted parasites, ongoing monitoring in these locations is warranted (*31*).

No gene-deleted parasites were observed within the cohorts from several countries (Cameroon, Gambia, Côte d’Ivoire, Madagascar, Malawi, Sierra Leone, South Africa, Sumatra, Uganda, Zimbabwe, and all countries in Asia); in 6 countries in Africa, a single gene-deleted parasite was found (*pfhrp2*: Ghana, Kenya, Mali, Togo, and Zambia; *pfhrp3*: Tanzania). The sample sizes are insufficient to comment on regional proportions. Baselines for these regions are undetermined, although a low level of false-negative results using HRP2-based RDTs has been reported in rural Ghana (*34*), and varying levels (0%–30%) of *pfhrp2* deletion have been observed in regions of Kenya (*12*). *pfhrp2/pfhrp3* double*-*deleted parasites have been observed within the China–Myanmar border area, where baseline *pfhrp2*-deleted parasite proportions were as low as 4% (*17*). The observation of a low level of gene-deleted parasites in this study emphasizes the need to monitor *pfhrp2/pfhrp3* status for early detection of emergent double deletions in the countries of origin.

SD BioLine and Carestart HRP2-based tests consistently produced the same outcome, but those results occasionally differed from results from BinaxNOW, which was less sensitive (Table 2). Subjecting the selected samples to testing with HRP2-based SD BioLine malaria RDTs corroborated the hypothesis that infections by *pfhrp2*-deleted parasites may occasionally fail to be detected, particularly in cases of low parasite density (<1,000/μL) and less-sensitive RDT varieties. Indeed, most *pfhrp2*-negative/*pfhrp3*-positive samples tested positive with HRP2-based SD BioLine malaria RDTs when parasitemia was >1000 parasites/μL, which suggests that HRP3 cross-reaction with HRP2-based tests acts as a failsafe, in cases of adequate parasite density (generally observed to be >1,000 parasites/μL [*14*]). *pfhrp2*-deleted parasites, in the absence of a double deletion, may suffer a loss of sensitivity, but these assays remain a viable interim option for remote and low-resource settings.

*P. falciparum* and pan–*Plasmodium* spp. RDTs failing to detect *pfhrp2/3*-deleted *P. falciparum* through pan–*Plasmodium* spp. pLDH likely reflects the freezing/thawing of whole blood samples, which is reported to degrade the antigen and to cause hemolysis of the blood, leading to sample inhibition (*35*). Freeze-thawing of archived blood is not observed to degrade HRP2 appreciably (*7*).

The main purpose of microsatellite analysis was to compare the genetic relatedness between parasites with gene deletions reported from different areas globally so that we could determine whether parasites with gene deletions were of de novo emergence. For this purpose, we used the same set of microsatellite markers that have been used in other parts of the world, including South America, and included a common control of 3D7 parasite in each run at different laboratories to calibrate outcomes. Microsatellite analyses found high heterogeneity of *P. falciparum* populations within and between Sudan, South Sudan, and Nigeria. The lack of genetic relatedness observed between gene-deleted parasites, including between the gene-deleted parasites observed in this study and those analyzed within Eritrea in a previous study (*10*), suggests independent, de novo emergence of parasites with gene deletions. The high level of genetic diversity may reflect the broad geographic and temporal sampling range, as well as the heterogenicity, of natural *P. falciparum* populations in areas with moderate or high transmission intensities.

We analyzed microsatellite peaks for the presence of multiple peaks (indicating multiple unique haplotypes within an individual infection). Three samples had evidence of infection by multiple strains with cocirculating strains potentially present at lower density; all other samples had no secondary peaks exceeding our thresholds for calling (this necessary threshold may prevent the detection of minor alleles). Although it detected few multiclonal infections in this sample set, microsatellite analysis was able to detect a very high level of heterogeneity (88 haplotypes/86 samples) within and between countries, demonstrating the quality of the analysis. The low level of multiple clone infections may reflect the source of samples. For instance, Nigeria is a high-transmission country with a high median multiplicity of infection; however, the Nigerian cohort was composed of travelers or immigrants to Australia who returned home for family events, usually traveling for <2 weeks.

A notable consideration when interpreting results from this study is the opportunity sampling. Using imported *P. falciparum* from travelers provided small sample sizes for most countries of origin and a broad collection timeframe (2010–2018). The sampling timeframe does not capture the true prevalence of *pfhrp2* and *pfhrp3* deletion in contemporary parasite populations or allow us to consider the effects of seasonal profiles (*38*). As a result, the cohort is not representative of cases within endemic regions, which is noteworthy because *pfhrp2/3* deletion needs to be interpreted considering clinical relevance. Deletion proportions between symptomatic and asymptomatic patients within groups were too small and too often status unknown for meaningful analysis. This limitation restricts the conclusions that can be drawn from the screening results, although analyses of imported malaria cases in persons entering Australia has the added benefit of informing local case management.

Because clinicians’ notes informed patient data, the specific geographic origins were limited to the country level (and, in 27 cases, were reported only as having origins in Africa). To the best of our knowledge, travelers contracted malaria parasites from their country of origin. However, we cannot exclude the possibility that parasites were contracted from another endemic region. Specimens from South Sudan were obtained primarily from refugees who had reported staying in camps for long periods (3–12 months), including settlements bordering Uganda and Ethiopia. Therefore, parasites may have originated from bordering endemic regions.

Malaria control is complicated in regions bordering other endemic nations by human–vector migration. Border regions are often rural, which may lead to high transmission coupled with inadequate health services (*36*). Similarly, the remoteness, limited resources, and political complexity of border regions often produces suboptimal surveillance responses (*37*). Given the genetic exchange expected between adjacent parasite populations, monitoring of *pfhrp2* and *pfhrp3* for Sudan and South Sudan would ideally be coordinated together with neighboring countries.

In conclusion, analysis of imported *P. falciparum* cases revealed *pfhrp2* and *pfhrp3* deletion from 12 countries, including levels of *pfhrp2*-deleted parasites exceeding 10% from Nigeria, Sudan, and South Sudan, where *pfhrp2-*based malaria RDT failure would constitute a major public health threat. These nations require urgent prevalence surveys and ongoing monitoring for early detection of emergent double deletion parasites.

Appendix Additional information on *Plasmodium falciparum* histidine-rich protein 2 and 3 gene deletions in strains from Nigeria, Sudan, and South Sudan.
