# Supplementary material for: Plasmodium falciparum Histidine-Rich Protein 2 and 3 Gene Deletions in Strains from Nigeria, Sudan, and South Sudan
Source: Emerg Infect Dis. 2021 Feb;27(2):471–9. doi: 10.3201/eid2702.191410 (PMC7853540; doi:10.3201/eid2702.191410)
Supplement: Appendix — Additional information on Plasmodium falciparum histidine-rich protein 2 and 3 gene deletions in strains from Nigeria, Sudan, and South Sudan. [file 19-1410-Techapp-s1.pdf]

Article DOI: https://doi.org/10.3201/eid2702.191410

Plasmodium falciparum Histidine-Rich Protein 2 and 3 Gene Deletions in Strains from Nigeria, Sudan, and South Sudan

Appendix

Appendix Table 1. Primers and PCR conditions: PCR conditions for assays investigating presence or absence of *pfhrp2* and *pfhrp3* exon1 and exon2, and assays amplifying 7 neutral microsatellite loci for genetic diversity and genetic relatedness analysis

Target	Primer	Primer sequence (5'-3')	PCR conditions	Reaction mixture (μL per reaction)	Expected product size	Reference
pfhrp2 exon2	*pfhrp2ex2*F<br>*pfhrp2ex2*R	CAA AAG GAC TTA ATT TAA ATA AGA G<br>AAT AAA TTT AAT GGC GTA GGC A	94°C/10min<br>[94°C/50s, 55°C/50s, 70°C/1min] × 45 cycles<br>72°C/10min	Total reaction volume: 25<br>(Template DNA: 10) + (10× PCR Buffer: 2.5) + (25 mM $MgCl_2$: 2) + (1.25 mM dNTPs: 4) + (Primer *pfhrp2ex2*F @75 μg/mL: 1.5) + (Primer *pfhrp2ex2*R @75 μg/mL: 1.5) + (Amplitaq Gold: 0.25) + (Molecular grade H_2O: 3.25)	600–1000 bp	(*1*)
pfhrp2 exon1-2	*2E12F1*<br>*2E12R1*	GGT TTC CTT CTC AAA AAA TAA AG<br>TCT ACA TGT GCT TGA GTA GTT TCG	94°C/10min<br>[94°C/50s, 55°C/50s, 60°C/1min] × 45 cycles<br>68°C/10min	Total reaction volume: 25<br>(Template DNA: 10) + (10× PCR Buffer: 2.5) + (25 mM $MgCl_2$: 2) + (1.25 mM dNTPs: 4) + (Primer *2E12F1* @75 μg/mL: 1.5) + (Primer *2E12R1* @75 μg/mL: 1.5) + (Amplitaq Gold: 0.25) + (Molecular grade H_2O: 3.25)	36 bp	(*2*)
pfhrp3 exon2	*pfhrp3ex2*F<br>*pfhrp3ex2*R	AAT GCA AAA GGA CTT AAT TC<br>TGC ATG ATG GGC ATC ACC TG	94°C/10min<br>[94°C/50s, 50°C/50s, 70°C/1min] × 45 cycles<br>72°C/7min	Total reaction volume: 25<br>(Template DNA: 10) + (10× PCR Buffer: 2.5) + (25 mM $MgCl_2$: 2) + (1.25 mM dNTPs: 4) + (Primer *pfhrp3ex2*F @75 μg/mL: 1.5) + (Primer *pfhrp3ex2*R @75 μg/mL: 1.5) + (Amplitaq Gold: 0.25) + (Molecular grade H_2O: 3.25)	250–600 bp	(*1*)
pfhrp3 exon1-2	*pfhrp3ex1-2*F<br>*pfhrp3ex1-2*R	TAT CCG CTG CCG TTT TTG CTT CC<br>TGC ATG ATG GGC ATC ACC TG	94°C/10min<br>[94°C/50s, 55°C/50s, 70°C/1min] × 45 cycles<br>72°C/10min	Total reaction volume: 25<br>(Template DNA: 10) + (10× PCR Buffer: 2.5) + (25 mM $MgCl_2$: 2) + (1.25 mM dNTPs: 4) + (Primer *pfhrp3ex1-2*F @75 μg/mL: 1.5) + (Primer *pfhrp3ex1-2*R @75 μg/mL: 1.5) + (Amplitaq Gold: 0.25) + (Molecular grade H_2O: 3.25)	303 bp	(*2*)
pfmsp1	O1 (F)<br>O2 (R)	CACATGAAAGTTATCAAGAACTTGTC<br>GTACGTCTAATTCATTTGCACG	94°C/10min<br>[94°C/25s, 50°C/35s, 68°C1min] × 45 cycles<br>68°C/10min	Total reaction volume: 25<br>(Template DNA: 10) + (10× PCR Buffer: 2.5) + (25 mM MgCl2: 2.5) + (2.5 mM dNTPs: 4) + (Primer *pfmsp1*O1 @50 μg/mL: 1) + (Primer *pfmsp1*O2 @50 μg/mL: 1) + (Amplitaq Gold: 0.25) + (Molecular grade H_2O: 3.75)	Variable	(*3*)
pfmsp2	S3 (F)	GAAGGTAATTAAAACATTGTC		Total reaction volume: 25	Variable	(*3*)

Target	Primer	Primer sequence (5'-3')	PCR conditions	Reaction mixture (μL per reaction)	Expected product size	Reference
	S2 (R)	GAGGGATGTTGCTGCTCCACAG	94°C/10min<br>[94°C/50s, 55°C/60s, 65°C/1min] × 45 cycles<br>65°C/10min	(Template DNA: 10) + (10× PCR Buffer: 2.5) + (25 mM MgCl2: 2.5) + (2.5 mM dNTPs: 4) + (Primer *pfmsp2*S3 @50 μg/mL: 1) + (Primer *pfmsp2*S2 @50 μg/mL: 1) + (Amplitaq Gold: 0.25) + (Molecular grade H_2O: 3.75)		
pfglurp	G4 (F)<br>G5 (R)	ACATGCAAGTGTTGATCC<br>GATGGTTTGGGAGTAACG	94°C/10min<br>[94°C/25s, 50°C/35s, 68°C/1min] × 45 cycles<br>68°C/10min	Total reaction volume: 25<br>(Template DNA: 10) + (10× PCR Buffer: 2.5) + (25 mM MgCl2: 2.5) + (2.5 mM dNTPs: 4) + (Primer *pfglurp*G4 @50μg/mL: 1) + (Primer *pfglurp*G5 @50μg/mL: 1) + (Amplitaq Gold: 0.25) + (Molecular grade H_2O: 3.75)	Variable	(*3*)
PolyA	Round 1:<br>PolyA-F<br>PolyA-R	AAA ATA TAG ACG AAC AGA<br>ATC AGA TAA TTG TTG GTA	94°C/5min<br>[94°C/30s, 42°C/30s, 40°C/30s, 65°C/40s] × 25 cycles<br>65°C/5min	Total reaction volume: 25<br>(Template DNA: 2) + (10× PCR Buffer no Mg: 1.5) + (25 mM $MgCl_2$: 1.8) + (1.25 mM dNTPs: 0.6) + (Primer F @10 μM: 0.6) + (Primer R @10 μM: 0.6) + (FastTaq: 0.25) + (Molecular grade H_2O: 7.7)	131–185 bp<br>(PET dye color: red)	(*4*)
	Round 2:<br>PolyA-2-F<br>PolyA-3IR	AAA ATA TAG ACG AAC AGA<br>***PET**-GAA ATT ATA ACT CTA CCA	94°C/5min<br>[94°C/30s, 42°C/30s, 65°C/30s] × 25 cycles<br>65°C/5min	Total reaction volume: 15<br>(Template DNA: 1) + (10× PCR Buffer no Mg: 1.5) + (25 mM $MgCl_2$: 1.5) + (1.25 mM dNTPs: 0.6) + (Primer F @10 μM: 0.6) + (Primer R @10μM: 0.6) + (FastTaq: 0.25) + (Molecular grade H_2O: 9)		
TA1	Round 1:<br>TA1-3F<br>TA1-R	CTA CAT GCC TAA TGA GCA<br>TTT TAT CTT CAT CCC CAC	94°C/5min<br>[94°C/30s, 42°C/30s, 40°C/30s, 65°C/40s] × 25 cycles<br>65°C/5min	Total reaction volume: 25<br>(Template DNA: 2) + (10× PCR Buffer no Mg: 1.5) + (25 mM $MgCl_2$: 1.8) + (1.25 mM dNTPs: 0.6) + (Primer F @10 μM: 0.6) + (Primer R @10μM: 0.6) + (FastTaq: 0.25) + (Molecular grade H_2O: 7.7)	162–189 bp<br>(6-FAM dye color: blue)	(*4*)
	Round 2:<br>TA1-2-R<br>TA1-2-R	***6FAM**-CCG TCA TAA GTG CAG AGC<br>TTT TAT CTT CAT CCC CAC	94°C/5min<br>[94°C/30s, 42°C/30s, 65°C/30s] × 25 cycles<br>65°C/5min	Total reaction volume: 15<br>(Template DNA: 1) + (10× PCR Buffer no Mg: 1.5) + (25 mM $MgCl_2$: 1.5) + (1.25 mM dNTPs: 0.6) + (Primer F @10 μM: 0.6) + (Primer R @10 μM: 0.6) + (FastTaq: 0.25) + (Molecular grade H_2O: 9)		
PK2	Round 1:<br>PFPK2-F<br>PFPK2-R	CTT TCA TCG ATA CTA CGA<br>CCT CAG ACT GAA ATG CAT	94°C/5min<br>[94°C/30s, 42°C/30s, 40°C/30s, 65°C/40s] × 25 cycles<br>65°C/5min	Total reaction volume: 25<br>(Template DNA: 2) + (10× PCR Buffer no Mg: 1.5) + (25 mM $MgCl_2$: 1.8) + (1.25mM dNTPs: 0.6) + (Primer F @10 μM: 0.6) + (Primer R @10μM: 0.6) + (FastTaq: 0.25) + (Molecular grade H_2O: 7.7)	162–183 bp<br>(NED dye color: black/yellow)	(*4*)
	Round 2:<br>PK2-2-F<br>PK2-2-R	TAG TAA CGA TGT TGA CAA<br>***NED**-AAA AAG GAG GAT AAA TAC AT	94°C/5min<br>[94°C/30s, 42°C/30s, 65°C/30s] × 25 cycles<br>65°C/5min	Total reaction volume: 15<br>(Template DNA: 1) + (10× PCR Buffer no Mg: 1.5) + (25 mM $MgCl_2$: 1.5) + (1.25 mM dNTPs: 0.6) + (Primer F @10μM: 0.6) + (Primer R @10 μM: 0.6) + (FastTaq: 0.25) + (Molecular grade H_2O: 9)		

Target	Primer	Primer sequence (5'-3')	PCR conditions	Reaction mixture (μL per reaction)	Expected product size	Reference
TA109	Round 1:<br>TA109_3-F<br>TA109_3-R	TAG GGA ACA TCA TAA GGA T<br><br>CCT ATA CCA AAC ATG CTA AA	94°C/5min<br>[94°C/30s, 42°C/30s, 40°C/30s, 65°C/40s] × 25 cycles<br>65°C/5min	Total reaction volume: 25<br>(Template DNA: 2) + (10× PCR Buffer no Mg: 1.5) + (25 mM $MgCl_2$: 1.8) + (1.25 mM dNTPs: 0.6) + (Primer F @10 μM: 0.6) + (Primer R @10 μM: 0.6) + (FastTaq: 0.25) + (Molecular grade H_2O: 7.7)	163–178 bp<br>(6-FAM dye color: blue)	(*4*)
	Round 2:<br>TA109-2-F<br>TA109-2-R	***FAM**-GGTTAAATCAGGACAACAT<br><br>CCT ATA CCA AAC ATG CTA AA	94°C/5min<br>[94°C/30s, 42°C/30s, 65°C/30s] × 25 cycles<br>65°C/5min	Total reaction volume: 15<br>(Template DNA: 1) + (10× PCR Buffer no Mg: 1.5) + (25 mM $MgCl_2$: 1.5) + (1.25 mM dNTPs: 0.6) + (Primer F @10 μM: 0.6) + (Primer R @10 μM: 0.6) + (FastTaq: 0.25) + (Molecular grade H_2O: 9)		
2490	Round 1:<br>2490-F<br>2490-R	TTC TAA ATA GAT CCA AAG<br><br>ATG ATG TGC AGA TGA CGA	94°C/5min<br>[94°C/30s, 42°C/30s, 40°C/30s, 65°C/40s] × 25 cycles<br>65°C/5min	Total reaction volume: 25<br>(Template DNA: 2) + (10× PCR Buffer no Mg: 1.5) + (25 mM $MgCl_2$: 1.8) + (1.25mM dNTPs: 0.6) + (Primer F @10 μM: 0.6) + (Primer R @10 μM: 0.6) + (FastTaq: 0.25) + (Molecular grade H_2O: 7.7)	69–83 bp<br>(FAM dye color: blue)	(*4*)
	Round 2:<br>2490-2-F<br>2490-2-R	TTC TAA ATA GAT CCA AAG<br><br>***FAM**-AGA ATT ATT GAA TGC AC	94°C/5min<br>[94°C/30s, 42°C/30s, 65°C/30s] × 25 cycles<br>65°C/5min	Total reaction volume: 15<br>(Template DNA: 1) + (10× PCR Buffer no Mg: 1.5) + (25 mM $MgCl_2$: 1.5) + (1.25 mM dNTPs: 0.6) + (Primer F @10 μM: 0.6) + (Primer R @10 μM: 0.6) + (FastTaq: 0.25) + (Molecular grade H_2O: 9)		

*Fluorescent labeled primers (boldface type) are light sensitive; exposure to light should be minimized. Microsatellite fluorescent primers were ordered through Applied Biosystems (https://www.thermofisher.com) as Custom 5' Fluorescent Labeled Oligo, 10,000 picomoles.

Appendix Table 2. Patient data and results summary for the study of *Plasmodium falciparum* histidine-rich protein 2 and 3 gene deletions

Continent	Country	Specimen voucher	Year of patient's birth	Sex	Parasitemia % RBC	Parasitemia Parasites/µL	Further Notes*	Year of collection	*pfhrp2* exon 1–2	*pfhrp2* exon 2	*pfhrp3* exon 1–2	*pfhrp3* exon 2	*pfmsp1/pfmsp2 /pfglurp*
Africa	Unknown	BDA55	1986	M	NA	NA	NA	2014	pos	pos	pos	pos	NA
		BDA57	1970	M	1.4	70,000	NA	2014	neg†	neg	pos	pos	pos/pos/pos
		BDA64	1989	M	<0.01	<500	NA	2014	pos	pos	pos	pos	NA
		BDA66	1961	M	1	50,000	NA	2013	pos	pos	pos	pos	NA
		BDA81	1948	M	0.9	45,000	NA	2013	pos	pos	pos	pos	NA
		BDA88	1978	M	0.9	45,000	NA	2012	pos	pos	pos	pos	NA
		BDA89	1974	M	<0.01	<500	NA	2012	pos	pos	pos	pos	NA
		BDA90	1945	F	1.2	60,000	NA	2012	pos	pos	pos	pos	NA
		BDA99	1964	M	0.46	23,000	NA	2012	pos	pos	pos	pos	NA
		BDA100	1974	M	<0.01	<500	NA	2012	pos	pos	pos	pos	NA
		BDB1	1955	M	2	100,000	NA	2012	neg	neg	pos	pos	pos/pos/pos
		BDB2	1958	M	11	550,000	NA	2012	pos	pos	pos	pos	NA
		BDB4	1964	M	NA	NA	NA	2012	pos	pos	pos	pos	NA
		BDB9	1992	M	1.1	55,000	NA	2012	neg	neg	pos	pos	pos/pos/pos
		BDB10	1977	M	<0.01	<500	NA	2012	pos	pos	pos	pos	NA
		BDB11	1948	F	<0.01	<500	NA	2011	neg	neg	pos	pos	pos/pos/pos
		BDB17	2004	M	NA	NA	NA	2011	pos	pos	pos	pos	NA
		BDB20	1981	F	2.4	120,000	NA	2011	pos	pos	pos	pos	NA
		BDB35	1959	M	<0.01	<500	NA	2011	pos	pos	pos	pos	NA
		BDB38	2006	M	0.3	15,000	NA	2010	pos	pos	pos	pos	NA
		BDB42	1990	F	<0.01	<500	NA	2010	pos	pos	pos	pos	NA
		BDB45	1965	M	0.04	2,000	NA	2010	pos	pos	pos	pos	NA
		BDB46	1980	M	0.01	500	NA	2010	pos	pos	pos	pos	NA
		BDB84	1993	M	2.2	110,000	NA	2017	pos	pos	pos	pos	NA
		BDB86	1976	M	<0.01	<500	NA	2017	pos	pos	pos	pos	NA
		BDB88	2008	F	0.4	20,000	NA	2017	pos	pos	pos	pos	NA
		BDB91	1982	M	NA	NA	NA	2017	pos	pos	pos	pos	NA
	Cameroon	BDA12	1957	M	4.13	206,500	P, R	2015	pos	pos	pos	pos	NA
		BDA13	1957	M	3.2	160,000	P, R	2015	pos	pos	pos	pos	NA
		BDA38	1974	M	<0.01	<500	NA	2014	pos	pos	pos	pos	NA
	Gambia	BDB33	1992	F	3.4	170,000	NA	NA	pos	pos	pos	pos	NA
		BDB36	1992	F	1.9	95,000	NA	2011	pos	pos	pos	pos	NA
		BDB39	2000	NA	0.15	7,500	NA	NA	pos	pos	pos	pos	NA
		BDB40	1949	NA	<0.01	<500	NA	NA	pos	pos	pos	pos	NA
		BDB41	1910	NA	0.03	1,500	NA	NA	pos	pos	pos	pos	NA
	Ghana	BDA85	1967	M	3.7	185,000	PNC	2012	pos	pos	pos	pos	NA
		BDA5	1967	F	<0.01	<500	P	2016	neg	neg	pos	pos	pos/pos/pos
		BDA45	2001	F	0.14	7,000	NA	2014	pos	pos	pos	pos	NA
		BDA46	1973	F	0.64	32,000	PNC	2014	pos	pos	pos	pos	NA
		BDA67	1983	M	0.12	6,000	PNC	2013	pos	pos	pos	pos	NA
		BDA70	1975	M	1.1	55,000	CM	2013	pos	pos	pos	pos	NA
		BDA96	1955	M	1.1	55,000	NA	20 12	pos	pos	pos	pos	NA
		BDA98	1955	M	1	50,000	NA	2012	pos	pos	pos	pos	NA
		BDB24	1972	M	<0.01	<500	NA	2011	pos	pos	pos	pos	NA
		BDB26	1966	M	4.27	213,500	NA	2011	pos	pos	pos	pos	NA

Continent	Country	Specimen voucher	Year of patient's birth	Sex	Parasitemia % RBC	Parasitemia Parasites/µL	Further Notes*	Year of collection	*pfhrp2* exon 1–2	*pfhrp2* exon 2	*pfhrp3* exon 1–2	*pfhrp3* exon 2	*pfmsp1/pfmsp2/pfglurp*
		BDB29	1967	F	0.1	5,000	P	2011	pos	pos	pos	pos	NA
		BDB37	1979	M	<0.01	<500	NA	2010	pos	pos	pos	pos	NA
		BDB51	1967	F	<0.01	<500	P	2016	pos	pos	pos	pos	NA
		BDB52	1967	F	6.7	335,000	NA	2016	pos	pos	pos	pos	NA
		BDB65	1980	M	0.3	15,000	PNC	2016	pos	pos	pos	pos	NA
		BDB80	1958	M	0.7	35,000	NA	2017	pos	pos	pos	pos	NA
		BDB100	1959	M	0.16	8,000	NA	2017	pos	pos	pos	pos	NA
	Ivory Coast	BDA75	1973	M	0.12	6,000	PNC	2014	pos	pos	pos	pos	NA
		BDA76	1987	F	0.1	5,000	CC	2014	pos	pos	pos	pos	NA
	Kenya	BDA42	1986	M	1.4	70,000	NA	2014	neg	neg	pos	pos	pos/pos/pos
		BDA58	1986	F	<0.01	<500	NA	2014	pos	pos	pos	pos	NA
		BDA61	1986	M	<0.01	<500	NA	2014	pos	pos	pos	pos	NA
		BDA62	2000	M	0.4	20,000	NA	2013	pos	pos	pos	pos	NA
		BDA63	1994	M	<0.01	<500	NA	2013	pos	pos	pos	pos	NA
		BDA71	1985	M	0.4	20,000	NA	2013	pos	pos	pos	pos	NA
		BDA72	2012	M	7.6	380,000	NA	2013	pos	pos	pos	pos	NA
		BDA73	1937	M	2.3	115,000	NA	2011	pos	pos	pos	pos	NA
		BDA74	1969	NA	NA	NA	NA	NA	pos	pos	pos	pos	NA
		BDB18	1969	NA	NA	NA	NA	NA	pos	pos	pos	pos	NA
		BDB19	1977	F	0.27	13,500	NA	2011	pos	pos	pos	pos	NA
		BDB21	1996	F	0.85	42,500	NA	2017	pos	pos	pos	pos	NA
		BDB30	1996	F	0.09	4,500	NA	2017	pos	pos	pos	pos	NA
		BDB98	1959	M	1	50,000	PNC	2014	pos	pos	pos	pos	NA
		BDC1	1974	M	<0.01	<500	PNC	2013	pos	pos	pos	pos	NA
		BDA59	1986	F	0.02	1,000	NA	2018	pos	pos	pos	pos	NA
		BDA83	1968	F	30.1	1,505,000	PNC	2016	pos	pos	pos	pos	NA
		BDC3	2003	F	1.5	75,000	NA	2016	pos	pos	pos	pos	NA
	Madagascar	BDA7	1984	F	<0.01	<500	NA	2017	pos	pos	pos	pos	NA
	Malawi	BDA6	1988	M	0.08	4,000	NA	2017	pos	pos	pos	pos	NA
		BDB77	2003	F	<0.01	<500	NA	2016	pos	pos	pos	pos	NA
		BDB79	NA	M	0.04	2,000	R	2010	pos	pos	pos	pos	NA
		BDA8	NA	F	1.5	75,000	R	2010	pos	pos	pos	pos	NA
		BDA9	NA	M	<0.01	<500	R	2010	pos	pos	pos	pos	NA
		BDA10	NA	F	<0.01	<500	R	2010	pos	pos	pos	pos	NA
	Mali	BDA14	1980	M	NA	NA	NA	2015	pos	pos	pos	pos	NA
		BDA18	1977	M	1.29	64,500	NA	2015	neg	neg	pos	pos	pos/pos/pos
		BDB62	1971	M	0.29	14,500	NA	2016	pos	pos	pos	pos	NA
	Nigeria	BDA11	1972	M	0.05	2,500	NA	2015	pos	pos	pos	pos	NA
		BDA15	1977	M	0.2	10,000	NA	2015	pos	pos	pos	pos	NA
		BDA17	1968	M	0.15	7,500	NA	2015	pos	pos	pos	pos	NA
		BDA24	1989	M	1.1	55,000	NA	2015	neg	neg	pos	pos	pos/pos/pos
		BDA25	1989	M	0.36	18,000	NA	2015	pos	pos	pos	pos	NA
		BDA29	1966	M	1.1	55,000	NA	2015	pos	pos	pos	pos	NA
		BDA44	1977	M	1.01	50,500	NA	2014	pos	pos	pos	pos	NA
		BDA48	1981	F	<0.01	<500	NA	2014	pos	pos	pos	pos	NA
		BDA60	1977	M	0.61	30,500	P	2014	pos	pos	pos	pos	NA
		BDA77	1982	M	<0.01	<500	NA	2013	pos	pos	pos	pos	NA

Continent	Country	Specimen voucher	Year of patient's birth	Sex	Parasitemia		Further Notes*	Year of collection	*pfhrp2* exon 1–2	*pfhrp2* exon 2	*pfhrp3* exon 1–2	*pfhrp3* exon 2	*pfmsp1/pfmsp2 /pfglurp*
					% RBC	Parasites/µL							
		BDA78	1976	M	NA	NA	NA	2013	pos	pos	pos	pos	NA
		BDA79	1976	M	1	50,000	NA	2013	pos	pos	pos	pos	NA
		BDA80	1976	M	<0.01	<500	NA	2013	pos	pos	pos	pos	NA
		BDA86	1968	M	<0.01	<500	P	2012	pos	pos	pos	pos	NA
		BDA87	1972	M	1.1	55,000	NA	2012	pos	pos	pos	pos	NA
		BDA91	1981	M	2.5	125,000	NA	2012	neg	neg	pos	pos	pos/pos/pos
		BDA92	1979	M	4	200,000	NA	2012	neg	neg	pos	pos	pos/pos/pos
		BDA93	1979	M	0.1	5,000	NA	2012	pos	pos	pos	pos	NA
		BDA94	1988	M	<0.01	<500	NA	2012	pos	pos	pos	pos	NA
		BDB7	1992	M	<0.01	<500	NA	2012	pos	pos	pos	pos	NA
		BDB8	1992	M	<0.01	<500	NA	2012	pos	pos	pos	pos	NA
		BDB16	1987	M	0.2	10,000	NA	2011	pos	pos	pos	pos	NA
		BDB22	1932	M	<0.01	<500	NA	2011	pos	pos	pos	pos	NA
		BDB23	1932	M	0.3	15,000	NA	2011	pos	pos	pos	pos	NA
		BDB25	1939	M	NA	NA	TF	2011	pos	pos	pos	pos	NA
		BDB31	1982	M	0.08	4,000	CC	2011	neg	neg	pos	pos	pos/pos/pos
		BDB44	1970	M	<0.01	<500	NA	2010	pos	pos	pos	pos	NA
		BDB58	1987	M	0.1	5,000	NA	2016	pos	pos	pos	pos	NA
		BDB72	1976	M	<0.01	<500	NA	2016	pos	pos	pos	pos	NA
		BDA65	1956	M	1	50,000	NA	2013	pos	pos	pos	pos	NA
	Sierra Leone	BDA43	1991	F	12.25	612,500	NA	2014	pos	pos	pos	pos	NA
		BDA49	1972	M	<0.01	<500	R	2014	pos	pos	pos	pos	NA
		BDA56	1992	M	0.1	5,000	NA	2014	pos	pos	pos	pos	NA
		BDA84	1966	F	0.2	10,000	NA	2013	pos	pos	pos	pos	NA
		BDB13	1994	NA	0.06	3,000	PNC	NA	pos	pos	pos	pos	NA
		BDB66	1969	M	<0.01	<500	NA	2016	pos	pos	pos	pos	NA
		BDB78	1971	M	<0.01	<500	NA	2017	pos	pos	pos	pos	NA
		BDB81	1982	F	0.33	16,500	NA	2017	pos	pos	pos	pos	NA
		BDB83	1972	M	0.15	7,500	NA	2017	pos	pos	pos	pos	NA
		BDB85	1978	M	<0.01	<500	NA	2017	pos	pos	pos	pos	NA
		BDB87	1989	F	0.38	19,000	NA	2017	pos	pos	pos	pos	NA
		BDB96	1979	M	0.05	2,500	NA	2017	pos	pos	pos	pos	NA
		BDC4	1986	M	0.5	25,000	NA	2018	pos	pos	pos	pos	NA
	South Africa	BDA40	1957	F	NA	NA	P	2014	pos	pos	pos	pos	NA
		BDB82	1959	F	<0.01	<500	NA	2017	pos	pos	pos	pos	NA
	South Sudan	BDA68	1993	M	0.4	20,000	NA	2013	pos	pos	pos	pos	NA
		BDA69	1993	M	1.5	75,000	NA	2013	pos	pos	pos	pos	NA
		BDA97	1945	F	0.03	1,500	NA	2012	pos	pos	pos	pos	NA
		BDB60	1970	M	0.15	7,500	NA	2016	pos	pos	pos	pos	NA
		BDB63	1983	M	<0.01	<500	NA	2016	pos	pos	pos	pos	NA
		BDB90	2000	F	<0.01	<500	R	2017	pos	pos	pos	pos	NA
		BDB94	1988	M	0.5	25,000	NA	2017	neg	neg	pos	pos	pos/pos/pos
		BDB95	1942	M	1.3	65,000	NA	2017	pos	pos	pos	pos	NA
		BDB97	1972	M	1.3	65,000	NA	2017	pos	pos	pos	pos	NA
		BDB99	1983	M	<0.01	<500	NA	2017	neg	neg	pos	pos	pos/pos/pos
		BDC99	2017	F	0.13	6,500	R	2018	pos	pos	pos	pos	NA
		BDD01	1988	M	0.26	13,000	R	2018	pos	pos	pos	pos	NA

Continent	Country	Specimen voucher	Year of patient's birth	Sex	Parasitemia % RBC	Parasitemia Parasites/µL	Further Notes*	Year of collection	*pfhrp2* exon 1–2	*pfhrp2* exon 2	*pfhrp3* exon 1–2	*pfhrp3* exon 2	*pfmsp1/pfmsp2 /pfglurp*
		BDD02	1975	M	0.05	2,500	R, CC	2018	pos	pos	pos	pos	NA
		BDD03	1980	M	0.5	25,000	R, CC	2018	pos	pos	pos	pos	NA
		BDD04	1991	M	1.24	62,000	R	2018	pos	pos	neg	neg	pos/pos/pos
		BDD05	1985	M	0.03	1,500	R	2018	pos	pos	pos	pos	NA
		BDC98	1964	F	0.50	25,000	R	2018	neg	neg	pos	pos	pos/pos/pos
	Sudan	BDA2	1996	M	0.79	39,500	P, TF	2016	neg	neg	pos	pos	pos/pos/pos
		BDA3	1995	M	0.3	15,000	P	2016	neg	neg	pos	pos	pos/pos/pos
		BDA4	1983	M	0.02	1,000	NA	2016	neg	neg	pos	pos	pos/pos/pos
		BDA16	1983	M	1.7	85,000	P	2015	pos	pos	pos	pos	NA
		BDA19	1975	F	0.2	10,000	P	2015	pos	pos	pos	pos	NA
		BDA21	1978	M	<0.01	<500	NA	2015	pos	pos	pos	pos	NA
		BDA22	1978	M	0.2	10,000	NA	2015	pos	pos	pos	pos	NA
		BDA23	1978	M	1.2	60,000	NA	2015	pos	pos	pos	pos	NA
		BDA26	1978	F	<0.01	<500	NA	2015	pos	pos	pos	pos	NA
		BDA27	1971	M	0.02	1,000	PNC	2015	pos	pos	pos	pos	NA
		BDA33	2007	F	1.29	64,500	NA	2015	pos	pos	pos	pos	NA
		BDA34	2007	F	0.6	30,000	NA	2015	pos	pos	pos	pos	NA
		BDA37	1946	M	<0.01	<500	NA	2014	neg	neg	pos	pos	pos/pos/pos
		BDA39	1946	F	NA	NA	TF	2014	pos	pos	pos	pos	NA
		BDA41	1970	M	0.06	3,000	NA	2014	pos	pos	pos	pos	NA
		BDA51	1976	F	0.67	33,500	NA	2014	pos	pos	pos	pos	NA
		BDA53	1979	M	0.09	4,500	NA	2014	pos	pos	pos	pos	NA
		BDA95	1957	M	3	150,000	NA	2012	pos	pos	pos	pos	NA
		BDB14	1977	M	0.5	25,000	PNC	2011	pos	pos	pos	pos	NA
		BDB15	2006	M	0.8	40,000	NA	2011	pos	pos	pos	pos	NA
		BDB27	1955	NA	NA	7,000	NA	NA	pos	pos	pos	pos	NA
		BDB28	1975	M	2	100,000	P	2011	pos	pos	pos	pos	NA
		BDB50	1974	M	0.63	31,500	NA	2010	pos	pos	pos	pos	NA
		BDB53	1975	M	3.7	185,000	PNC	2016	pos	pos	pos	pos	NA
		BDB54	1975	M	0.63	31,500	NA	2016	pos	pos	pos	pos	NA
		BDB55	1996	M	NA	NA	NA	NA	pos	pos	pos	pos	NA
		BDB56	1976	M	0.07	3,500	NA	2016	pos	pos	pos	pos	NA
		BDB57	1976	M	0.5	25,000	NA	2016	pos	pos	pos	pos	NA
		BDB64	1961	M	0.6	30,000	CC	2016	pos	pos	pos	pos	NA
		BDB67	1984	M	0.8	40,000	NA	2016	pos	pos	pos	pos	NA
		BDB68	1988	M	0.02	1,000	NA	2016	pos	pos	pos	pos	NA
		BDB70	1992	F	<0.01	<500	NA	2016	pos	pos	pos	pos	NA
		BDB71	1947	M	<0.01	<500	NA	2016	pos	pos	pos	pos	NA
		BDB73	2011	M	1.3	65,000	NA	2016	pos	pos	pos	pos	NA
		BDB74	1998	F	<0.01	<500	NA	2016	pos	pos	pos	pos	NA
		BDB75	2010	F	0.5	25,000	NA	2016	pos	pos	pos	pos	NA
		BDB76	1977	M	0.1	5,000	NA	2016	pos	pos	pos	pos	NA
		BDB89	2001	M	<0.01	<500	NA	2017	pos	pos	pos	pos	NA
		BDC5	2001	F	2	100,000	NA	2018	pos	pos	neg	neg	pos/pos/pos
	Sumatra	BDA36	1965	M	1.7	85,000	NA	2014	pos	pos	pos	pos	NA
		BDC2	1968	M	0.1	5,000	NA	2017	pos	pos	neg	neg	pos/pos/pos
	Tanzania	BDA28	1968	M	<0.01	<500	CC	2015	neg	neg	pos	pos	pos/pos/pos

Continent	Country	Specimen voucher	Year of patient's birth	Sex	Parasitemia % RBC	Parasitemia Parasites/µL	Further Notes*	Year of collection	*pfhrp2* exon 1–2	*pfhrp2* exon 2	*pfhrp3* exon 1–2	*pfhrp3* exon 2	*pfmsp1/pfmsp2 /pfglurp*
		BDA47	1971	M	<0.01	<500	NA	2014	pos	pos	pos	pos	NA
		BDB61	1990	M	<0.01	<500	NA	2016	pos	pos	pos	pos	NA
		BDA82	1985	M	0.9	45,000	PNC	2013	pos	pos	pos	pos	NA
	Togo	BDA50	1983	M	0.27	13,500	NA	2014	neg	neg	pos	pos	pos/pos/pos
	Uganda	BDA54	1984	M	1.07	53,500	NA	2014	pos	pos	pos	pos	NA
		BDB47	1980	M	1.2	60,000	NA	2010	pos	pos	pos	pos	NA
	Zambia	BDA31	1958	M	0.2	10,000	NA	2015	neg	neg	pos	pos	pos/pos/pos
		BDB3	1960	M	<0.01	<500	NA	2012	pos	pos	pos	pos	NA
		BDB32	1956	F	0.04	2,000	NA	2011	pos	pos	pos	pos	NA
		BDB59	1939	F	8	400,000	NA	2016	pos	pos	pos	pos	NA
		BDB92	1943	M	0.6	30,000	P	2017	pos	pos	pos	pos	NA
	Zimbabwe	BDA1	1975	F	0.16	80,00	NA	2016	pos	pos	pos	pos	NA
Asia	Cambodia	BDC6	1971	F	3.3	165,000	TF	2018	pos	pos	pos	pos	NA
	India	BDB48	1940	M	<0.01	<500	NA	2010	pos	pos	pos	pos	NA
		BDB49	1940	M	0.1	5,000	NA	2010	pos	pos	pos	pos	NA
		BDB69	2011	F	3	150,000	NA	2016	pos	pos	pos	pos	NA
	Indonesia	BDB43	1958	M	2	100,000	NA	2010	pos	pos	pos	pos	NA
	Papua New Guinea	BDA30	1958	M	<0.01	<500	P, TF	2015	pos	pos	pos	pos	NA
		BDA35	1986	F	0.3	15,000	NA	2015	pos	pos	pos	pos	NA
		BDB5	1996	M	<0.01	<500	NA	2012	pos	pos	pos	pos	NA
		BDB12	1973	M	0.7	35,000	NA	2011	pos	pos	pos	pos	NA
		BDB34	1991	NA	0.8	40,000	NA	NA	pos	pos	pos	pos	NA
	Thailand	BDA20	1981	M	0.19	9,500	NA	2015	pos	pos	pos	pos	NA
South America	Peru	BDA52	1986	F	0.4	20,000	CC	2014	pos	pos	neg	neg	pos/pos/pos

* CC, reported concurrent condition; NA, not applicable; neg, negative; P, prophylaxis compliant; PNC, prophylaxis noncompliant; pos, positive; R, known refugee status; TF, previous treatment failure.
†Green shading indicates negative results for the target from both assays.

References

<jrn>1. Baker J, McCarthy J, Gatton M, Kyle DE, Belizario V, Luchavez J, et al. Genetic diversity of *Plasmodium falciparum* histidine-rich protein 2 (PfHRP2) and its effect on the performance of PfHRP2-based rapid diagnostic tests. J Infect Dis. 2005;192:870–7. PubMed https://doi.org/10.1086/432010</jrn>

<jrn>2. Gamboa D, Ho MF, Bendezu J, Torres K, Chiodini PL, Barnwell JW, et al. A large proportion of *P. falciparum* isolates in the Amazon region of Peru lack *pfhrp2* and *pfhrp3*: implications for malaria rapid diagnostic tests. PLoS One. 2010;5:e8091. https://doi.org/10.1371/journal.pone.0008091</jrn>

<jrn>3. Cheng Q, Gatton ML, Barnwell J, Chiodini P, McCarthy J, Bell D, et al. *Plasmodium falciparum* parasites lacking histidine-rich protein 2 and 3: a review and recommendations for accurate reporting. Malar J. 2014;13:283. https://doi.org/10.1186/1475-2875-13-283</jrn>

<jrn>4. Berhane A, Anderson K, Mihreteab S, Gresty K, Rogier E, Mohamed S, et al. Major threat to malaria control programs by *Plasmodium falciparum* lacking histidine-rich protein 2, Eritrea. Emerg Infect Dis. 2018;24:462–70. https://doi/org/10.3201/eid2403.171723</jrn>

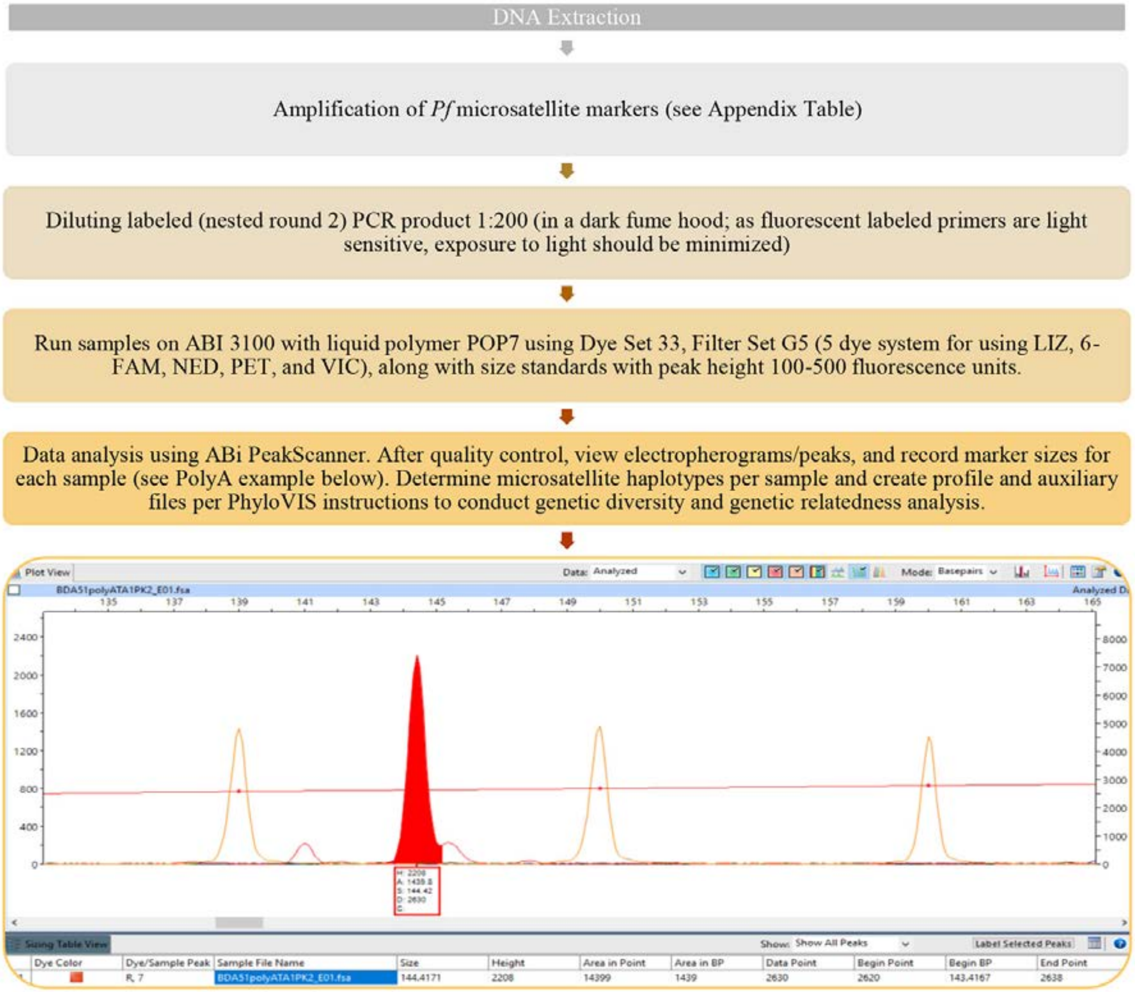

Appendix Figure 1. Workflow for *P. falciparum* microsatellite analysis.

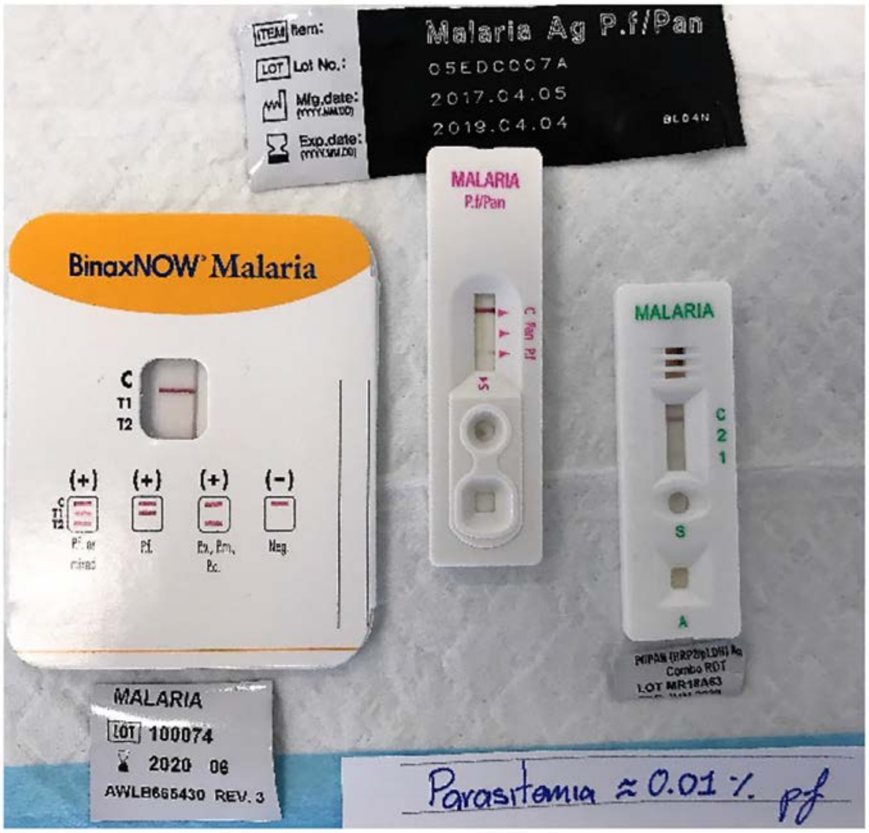

Appendix Figure 2. Results for sample BDB99 (originating from South Sudan, 2017, <0.01% parasitemia, *pfhrp2* negative/*pfhrp3* positive) using BinaxNOW, CareStart, and BioLine HRP2-based RDTs. CareStart and SD BioLine show no pan *spp.* band and a faint (1) *pf* band. BinaxNOW shows a negative pan *spp.* and *pf* outcome. Testing was conducted at Westmead Institute for Medical Research, Westmead, Australia, on January 4, 2019.

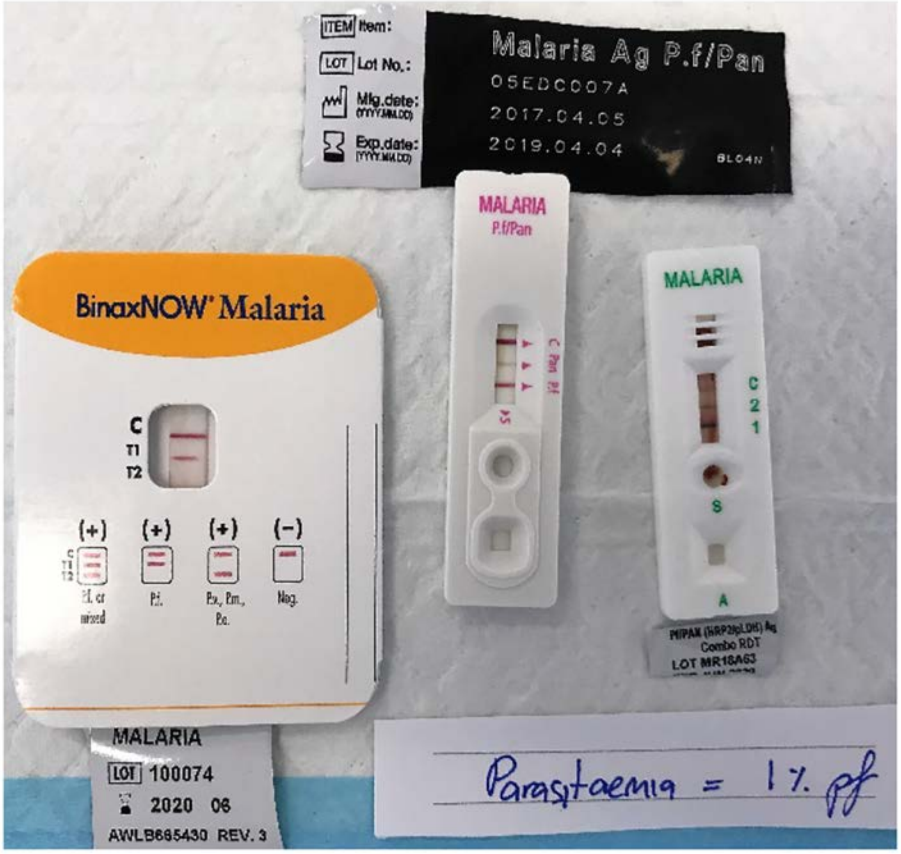

Appendix Figure 3. Results for sample BDD4 (originating from South Sudan, 2018, 1.24% parasitemia, *pfhrp2* positive/*pfhrp* negative) using BinaxNOW, CareStart, and BioLine HRP2-based RDTs. CareStart and SD BioLine show a faint (1) pan *spp.* band and a strong (4) *pf* band. BinaxNOW shows a negative pan *spp.* outcome and a moderate (2) *pf* band. Testing was conducted at Westmead Institute for Medical Research, Westmead, Australia, on January 4, 2019.
